# A Distributed Reasoning Engine Ecosystem for Semantic Context-Management in Smart Environments

**DOI:** 10.3390/s120810208

**Published:** 2012-07-30

**Authors:** Aitor Almeida, Diego López-de-Ipiña

**Affiliations:** Deusto Institute of Technology (DeustoTech), University of Deusto, Bilbao 48007, Spain; E-Mail: dipina@deusto.es

**Keywords:** intelligent environments, context-aware systems, semantic inference, multi-agent systems, distributed reasoning

## Abstract

To be able to react adequately a smart environment must be aware of the context and its changes. Modeling the context allows applications to better understand it and to adapt to its changes. In order to do this an appropriate formal representation method is needed. Ontologies have proven themselves to be one of the best tools to do it. Semantic inference provides a powerful framework to reason over the context data. But there are some problems with this approach. The inference over semantic context information can be cumbersome when working with a large amount of data. This situation has become more common in modern smart environments where there are a lot sensors and devices available. In order to tackle this problem we have developed a mechanism to distribute the context reasoning problem into smaller parts in order to reduce the inference time. In this paper we describe a distributed peer-to-peer agent architecture of context consumers and context providers. We explain how this inference sharing process works, partitioning the context information according to the interests of the agents, location and a certainty factor. We also discuss the system architecture, analyzing the negotiation process between the agents. Finally we compare the distributed reasoning with the centralized one, analyzing in which situations is more suitable each approach.

## 1. Introduction

Smart environments need to know the context information [1] in order to react to the changes in it. Managing correctly the context data allows applications to adapt themselves better to it, providing an enhanced experience to the users. Usually ontologies are regarded as one of the best approaches [2] to model the context information. OWL [3] ontologies offer a rich expression framework to represent the context data and its relations. In our previous works developing a context management middleware [4] we encountered significant problems when using semantic reasoning engines in real smart environments. As the number of triples in the ontology increases the inference time for environment actions becomes unsustainable. In order to be able to deploy these systems in real environments they must be able to react to context changes is a reasonable time for the reaction to be considered appropriate. On the other hand, modern smart environments host a diverse ecosystem of computationally enabled devices. With the wide adoption of low-cost platforms like Arduino (http://www.arduino.cc/), Teensy (http://www.pjrc.com/teensy/), Raspberry Pi (http://www.raspberrypi.org/) and STM32 Discovery (http://es.mouser.com/stm32discovery/) and the ubiquity of the smartphones, Smart Environments [5] have at their disposal nowadays more pervasive computing capabilities than ever. The existence of a large number of devices available in the environment offers several advantages to help context management systems overcome the problem of high inference time in semantic reasoning.

In our approach, we have decided to split the inference problem among different reasoning engines or context consumers according to the interests stated by each of them. The inference is no longer performed by a central reasoning engine, but divided into a peer-to-peer network of context producers and context consumers. Dividing the inference problem into smaller sub-units makes it more computationally affordable. Context consumers only receive the information they are interested in and do not have to process non-relevant data. Another benefit is that the different parts of the reasoning process can take place in parallel, reducing the global time taken to resolve the inference problem. This parallelization allows us to achieve a more feasible response time in the reactions to environmental changes. In order to create this distributed architecture we have used a multi-agent solution were each agent represent a context provider (sensor data, inferred facts from the reasoning engine) or a context consumer (reasoning engines, actuators that react to environmental change). Each of these agents has a set of interests, represented by the context data type, a location and the certainty factor of the data. Taking into account this information agents organize themselves using the Contract Network Protocol into an architecture that allows them to satisfy their interests.

In this paper we present the proposed system. In Section 2 we analyze the related work in distributed reasoning and describe several uses of the Contract Network Protocol; in Section 3 we describe the inference sharing process, explaining how the inference is split into smaller sub-units; in Section 4 we discuss the system architecture, analyzing the negotiation process; in Section 5 we describe the validation process and discuss the obtained results, identifying the factors that influence the performance of the distributed reasoning and finally in Section 6 we expose the conclusions and describe the future work.

## 2. Related Work

Several authors have tackled the problem of the inference distribution in scenarios where the knowledge is not centralized. This decentralization can occur for several reasons:

- The nature of the selected domain. For example the peer-to-peer architecture of the sensor networks or the distribution of knowledge in the Semantic Web.
- The need to distribute the reasoning to be able to process large sets of data.

In [6] the authors present an algorithm that addresses the problem of reasoning with multiple related ontologies. Their system interconnects multiple OWL ontologies, using mappings between concepts and applies a distributed tableau reasoning technique. Their approach is to create a distributed algorithm while we share the knowledge between a network of inference engines based on their declared interests.

In [7] the authors present a parallel and distributed platform for processing large amounts of distributed RDF data. They discuss a divide-conquer-swap strategy that converges towards completeness. The differences with our approach are two: (1) we split the problem in loosely coupled subparts while they see it as a complete problem and (2) they do the fact sharing process automatically while in our case this process is dictated by the previously expressed interests of each context consumer. In [8] the authors present a consequence finding algorithm for peer-to-peer settings. The algorithm computes consequences gradually from the solicited peer to peers that are increasely distant. Authors argue that centralizing the reasoning in a single server in peer-to-peer systems is not feasible for two reasons: first it would be costly to gather the data available through the system and second it would be useless because of the dynamicity of these types of networks, where peers can join and leave the system. In [9] the authors introduce an architecture for designing distributed inference algorithms in ad hoc sensor networks. The architecture is centered in solutions for sensor networks and takes into account the movement of the nodes. In [10] a Java package that provides programmers with a middleware that allows to ensemble different inference systems implemented in Jess transparently is presented. Like our architecture the proposed middleware offers temporal and spatial decoupling of the inference, but their middleware is linked to one specific reasoner, Jess. In our case, due to the usage of OWL ontologies to model the context, different semantic reasoning engines can be used in each context consumer seamlessly. In [11] the authors introduce an architecture for distributed inference in sensor networks. This architecture is robust to unreliable communication and node failures. To do this the nodes of the network assemble in a stable spanning tree, and later transform the spanning tree into a junction tree for the inference problem. Finally in [12] the authors present a distributed algorithm for approximate probabilistic inference in dynamical systems. This distributed approach to approximate dynamic filtering is based on a distributed representation of the assumed density in the network.

Our approach differs to the previously discussed systems in the following aspects:

- It is not based on an ad hoc distributed inference algorithm. We split the reasoning problem in different loosely coupled subparts according to the interests expressed by the context consumers. This allows us to use standard modeling techniques (OWL ontologies) and reasoning engines (in our case semantic reasoning engines like Pellet [13] or Jena 2 Inference Support [14]).
- We take into account the certainty factor of the data. This allows us to filter low quality data, reducing the inference time lost in useless reasoning.
- We also take into account the location of the context data in order to filter the information received by the Context Consumers.

The Contract Net Protocol (CNP) is a widely used protocol in multi-agent systems. It has proved to be a flexible and low communication interaction protocol for task assignment [15]. CNP has been used since the early 80 s [16,17] and it has undergone several improvements since then [18], like the ones described in [19–21]. Multi-agent systems based on the Contract Net Protocol have been used to tackle several problems. In [22] the authors discuss a system for efficient job scheduling on the Grid using multi-agent systems and a Service Level Agreement (SLA) negotiation protocol based on the Contract Net Protocol. The proposed system is composed by three types of agents: user agents, local scheduler agents, and super scheduler agents. In [23] the authors propose a multi-agent system with Petri nets to design and implement holonic manufacturing systems (HMS) to fulfill the requirements of orders. Authors describe an architecture and a two-layer contract net protocol for planning order holons, product holons and resource holons in HMS. In [24] the authors use a multi-agent system based on contract net protocol for reservoir flood control optimization. Finally in [25,26] the authors describe a multi-agent architecture for dynamic scheduling in flexible manufacturing systems which involves only resource agents using contract net protocol.

## 3. Inference Sharing Process

**Figure 1.**
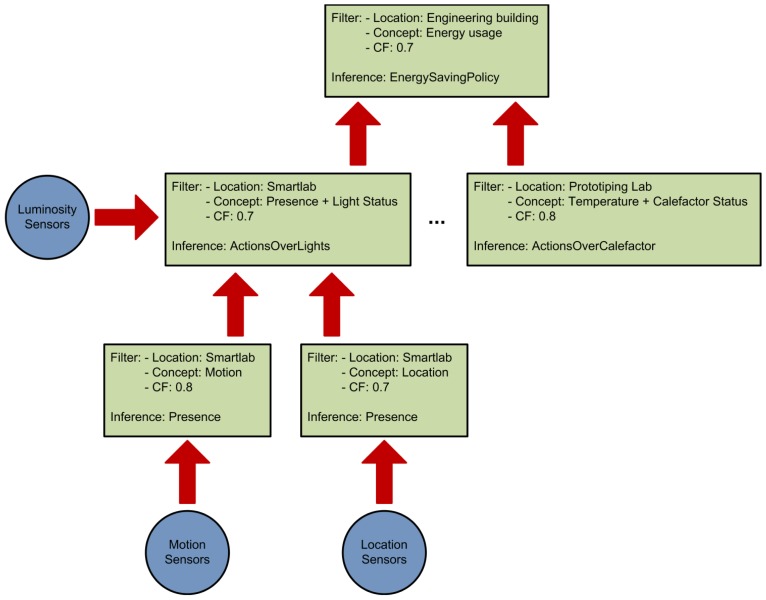
Reasoning hierarchy depicting the sensors (circles) and reasoning engines (squares) that take part in the inference process.

Our system relies on splitting the reasoning process (see Figure 1) into smaller inference units in order to achieve the following results:

1. Attain the temporal decoupling of the different inference units. This will allow the inference to be done concurrently in various reasoning engines. The parallelization of the inference process reduces the time required to reach certain conclusions.
2. Attain the spatial decoupling of the inference process. This will increase the general robustness of the system making it more fault-tolerant. Problems in one reasoning engine will not stop all the inference, as opposed to a centralized approach
3. Reduce the number of triples and rules that each reasoning engine has to manage. This allows the use of more computationally constrained devices to carry out the inference process.
4. Compartmentalize the information according to the different interests of the reasoning engines. This will reduce the amount of data that is shared over the network.
5. Allow the dynamic modification of the created organization. Devices in modern smart environments can change their location frequently (e.g., mobile phones, mote). The created hierarchy must change to adapt itself to these modifications.

This organization also allows the creation of a reasoning engine hierarchy that provides different abstraction levels in the inferred facts. As can be seen in Figure 1 the reasoning engines at the bottom use pretty simple concepts. But as the reasoning ascends in the hierarchy the concepts become more complex and abstract as a result of the aggregation of the different inferred facts.

**Figure 2.**
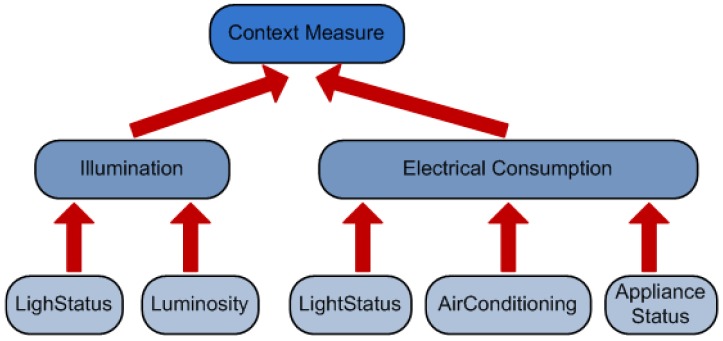
Part of the concept taxonomy extracted from the AMBI2ONT ontology.

**Figure 3.**
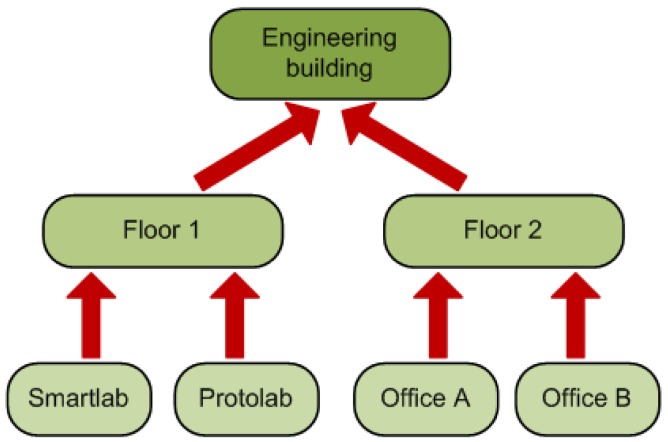
Part of the location taxonomy used on our system. The taxonomy depicts the “contains” relations of the used ontology.

In order to decide how the reasoning should be divided among the reasoning engines we take into account three factors:

1. *The ontological concepts that will be used by the reasoner.* These concepts are organized in a taxonomy (see Figure 2) depicting the class relations of the ontology. In our case we use the AMBI^2^ONT [27] ontology developed in our previous research and explained in the next subsection. When trying to find an appropriate context provider the context consumer can search for specific concepts (in our example “Luminosity”) or broader ones (as “Electrical Consumption” that encompass “LightStatus”, “AirConditioning” and “ApplianceStatus”).
2. *The location where the measures originate from.* As with the ontological concepts we have a taxonomy of the locations extracted from our ontology. This taxonomy models the “contains” relations between the different rooms, floors and buildings (see Figure 3). The context consumer can search for an specific location (the Smartlab laboratory in our example) or for a set of related locations (for example all the rooms in the first floor of the engineering building).
3. *The certainty factor (CF) associated to each ontological concept.* As we discussed in [27] modeling real environments taking certainty for granted is usually a luxury that a context management framework cannot afford. Reality, and so the context, is ambiguous. Sensors and devices are not perfect and their measures carry a degree of uncertainty, several thermometers in the same room can provide conflicting measures of the temperature. For this reason the context consumer can specify a minimum CF in its searches. This CF model is the one described in [28] and not the original CF that takes values between −1 and 1 previously described in [29]. Detailed information about the reasoning using the CF can be found in Section 4 of [27].

An example of the usage of these three factors to divide the reasoning process can be seen in Figure 1. The different context consumers state their requirements (green squares in the figure) and a reasoning hierarchy is created according to them.

### 3.1. The AMB^2^ONT Ontology

**Figure 4.**
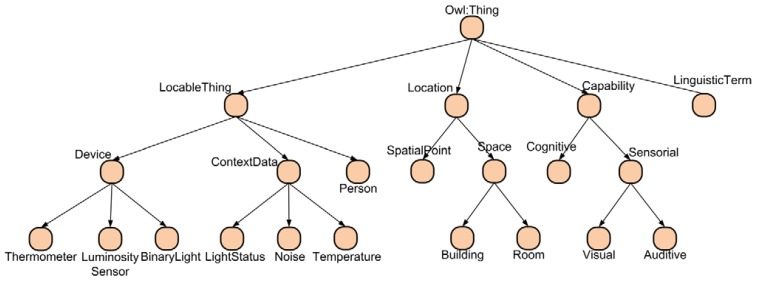
Subset of the main ontology concepts. Image extracted from [27].

The AMB^2^ONT was developed in a previous work to model the ambiguity in contextual information. In this subsection we will briefly describe the used ontology. A more detailed description of the ontology is given in [27] along with an in depth explanation on how vagueness and uncertainty interact with semantic context inference. In our case we consider two aspects of the ambiguity: the uncertainty and the vagueness. We use uncertainty to model the truthfulness of the different context data by assigning to them a certainty factor (CF). On the other hand, vagueness helps us to model those situations where the boundaries between categories are not clearly defined. This usually occurs when users are involved. The main elements of the ontology are:

- *Location*: The subclasses of this class represent the location concepts of the context. In our system we have three types of locations: points, rooms and buildings.
- *LocableThing*: The subclasses of this class represent the elements of the system that have a physical location. It contains three subclasses: the Person subclass represents the users, the Device subclass models the different devices of the environment and the *ContextData* subclass models the measures taken by the sensors. As we will explain in the next section there are two types of measures, those taken by the devices and the global measures for each room calculated by our data fusion mechanism. Figure 4 shows a subset of the type of context data taken into account in the ontology.
- *LinguisticTerm*: This class models the fuzzy linguistic terms of the values of the context data. The ontology only stores the linguistic term and membership value of each individual of context data. Currently the ontology does not model the membership functions and rules used by the inference engine.
- *Capability*: The subclasses of this class model the capabilities of users and their mobile devices.

Each ContextData individual has the following properties:

- *crisp_value*: the measure taken by the associated sensor. In our system a sensor is defined as anything that provides context information.
- *certainty_factor*: the degree of credibility of the measure. This metric is given by the sensor that takes the measure and takes values between 0 and 1.
- *linguistic_term*: each measure has its fuzzy representation, represented as the linguistic term name and the membership degree for that term.

**Figure 5.**
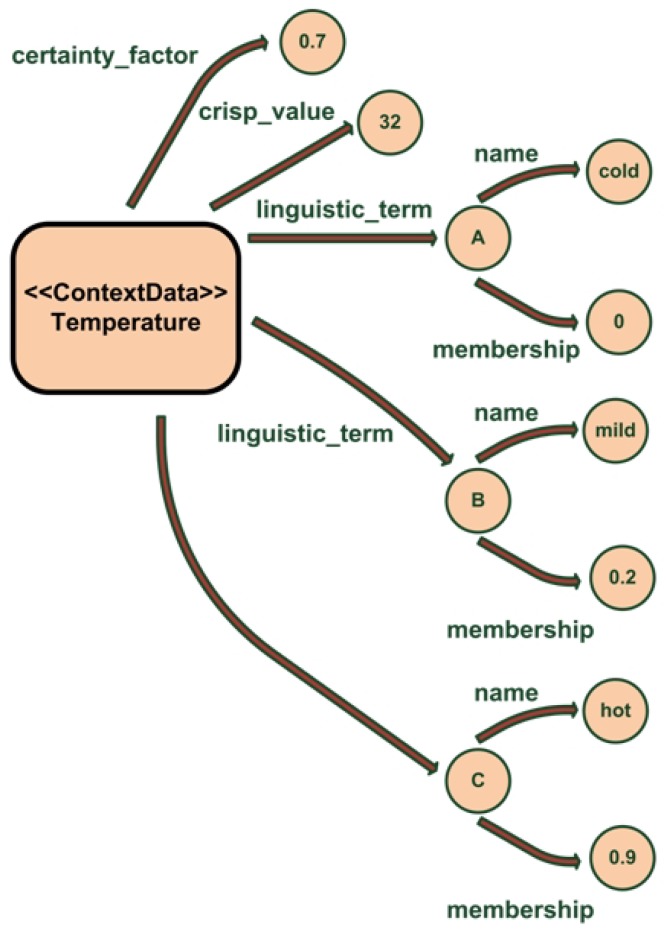
Example of the ambiguity data for a temperature measure stored in the ontology. Image extracted from [27].

This can be seen in the example shown on Figure 5. The temperature measure has a crisp value of 32 °C with a certainty factor of 0.7. After processing that crisp value with the associated membership functions our system has inferred that the membership degree for cold is 0, for mild is 0.2 and for hot is 0.9; so the room is mainly hot.

## 4. System Architecture

**Figure 6.**
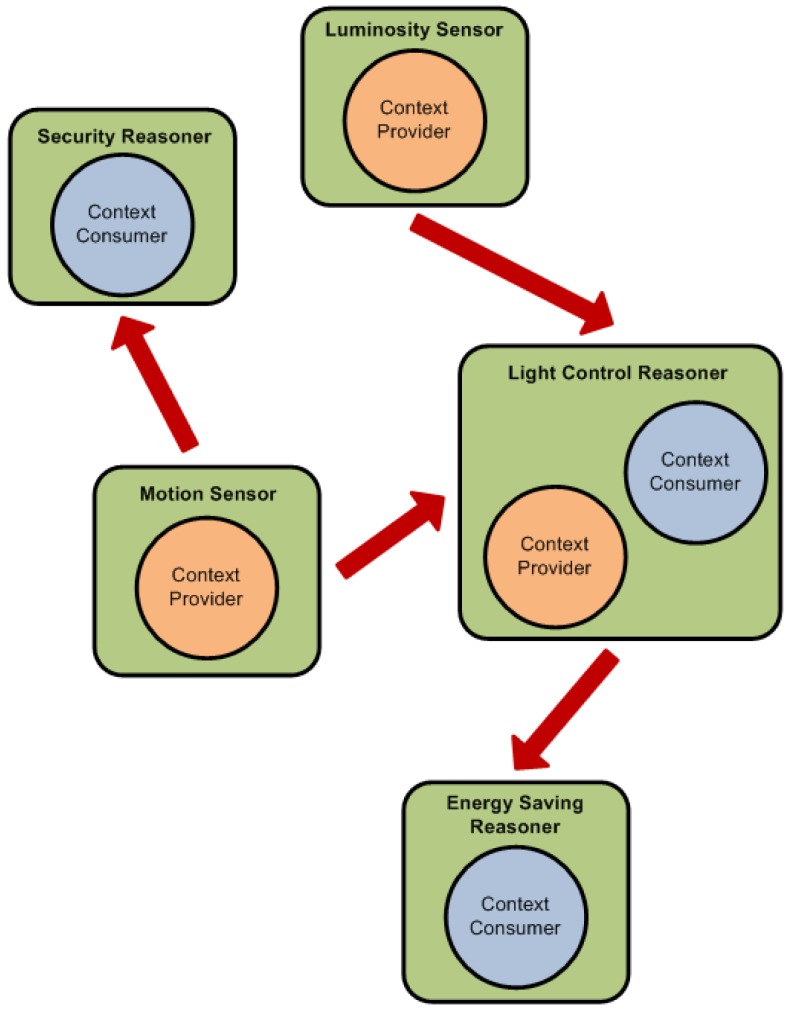
Example of the agents in the architecture.

To create this hierarchy we have used an agent-based architecture (see Figure 6) with two types of agents:

1. *Context Providers:* These agents represent those elements in the architecture that can provide any context data. Each context provider has one context type (luminosity, temperature…), one location and a certainty factor. If a device can provide more than one context type then it will be represented by more one agent for each context type. Context Providers can be sensors, devices or inference engines that provide the inferred facts to other Context Consumers.
2. *Context Consumers:* These agents represent those elements that need to consume context data. Each context consumer has a set of interests defined by the context types, locations and minimum certainty factors. Context Consumers can be inference engines, devices or actuators that react to the changes in the environment.

**Figure 7.**
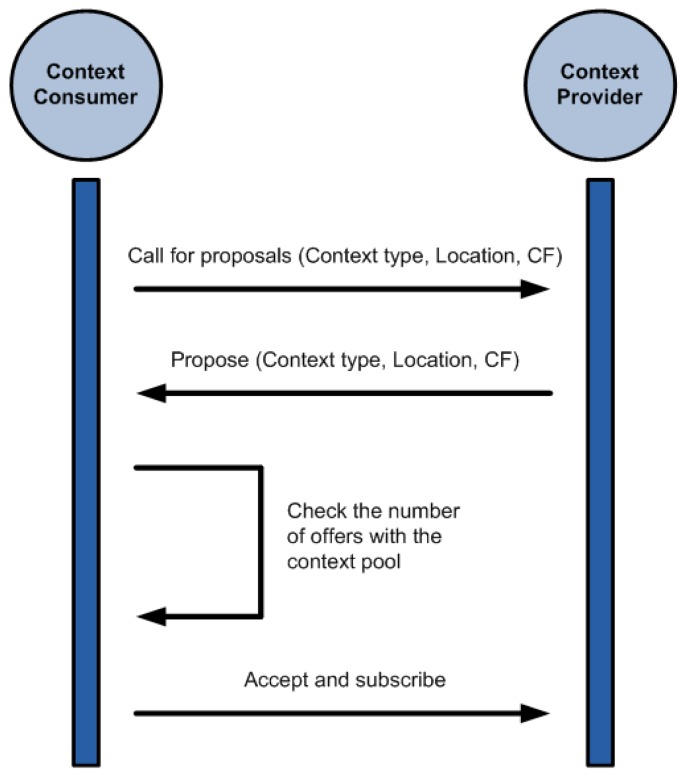
Negotiation between a context provider and a context consumer.

The differentiation between providers and consumers is simply logical. The same device can act as a provider and a consumer at the same time, receiving data from sensors and supplying the inferred facts to another device. While the resulting inference process follows somewhat a hierarchy, the agent structure is purely a peer-to-peer architecture where there is no central element. Agents negotiate between them to find which other agents can meet their needs. This process can be seen in Figure 7.

The architecture has been implemented using the JADE framework [30]. Java Agent DEvelopment Framework (JADE) is a software framework implemented in Java. It simplifies the implementation of multi-agent systems through a middleware that comply with the FIPA (http://www.fipa.org/) (Foundation for Intelligent Physical Agents) specifications. JADE provides several tools that facilitate the development of multi-agent system:

1. A *runtime environment* where agents reside.
2. A *library* that allow developers to create their agents.
3. *Tools to administer and control* the deployed agents.

A JADE application is composed of multiple *Agents*, each one having its own unique identifier. Each agent executes different tasks and communicates with other agents exchanging messages. Agents exists on top of a *Platform*, which provides to the agents a series of services (like the message delivering one). A platform is composed by *Containers*. Each running instance of the JADE runtime environment is called a container as it can contain several agents. Containers can be run in different machines, achieving so the distributed platform. There is always one *Main Container* in each platform and all other containers register in it. The main container differs from other containers in that it must be the first container to start in the platform and that it includes two special agents:

1. *AMS (Agent Management System)*: provides the naming service and is the only agent able to manage the platform (start, kill, shutdown agents, create remote containers). Other agents must send requests to the AMS to perform these actions.
2. *DF (Directory Facilitator)*: provides a Yellow Pages service where agents can announce the services they provide or find the services provided by other agents.

Communication in JADE is based in the asynchronous message passing paradigm. Agents can communicate between themselves no matter the container or platform they reside in. Each agent has a message queue where the JADE runtime posts the messages sent by other agents. The main components of a message are:

- The *sender* of the message.
- The *receivers* of the message.
- The communicative intention. It is called the *performative* and indicates what the intention of the sender is. Examples of performatives are: REQUEST, INFORM, ACCEPT_PROPOSAL, QUERY_IF.
- The *content* with the information of the message.

### The Negotiation Process

To be able to take part in the negotiation process all participants have to have information about the three factors described in Section 3. Without this data the negotiation process cannot happen. The negotiation follows these steps:

1. The *Context Consumer Agent (CCA)* sends a *Call For Proposals (CFP)* stating the context type and location that it is interested in and the minimum *certainty factor (CF)* expected from the context.
2. The *Context Provider Agents (CPA)* replies to the CFP with individual proposals stating what they can offer to the CCA.
3. The CCA checks the received proposals. Some of the context consumers have a maximum number of context sources they can subscribe to concurrently. This is dictated by the computational capabilities of the device running the CCA. If the number of received proposals is above this limit the CCA selects those that it considers better (comparing their CF).
4. The CCA sends an Accept to the selected CCPs and subscribes to the context updates.

The negotiation meets the FIPA ACL specification [31] for agent communication, following the Contract Net Protocol [16].

## 5. Evaluation

**Figure 8.**
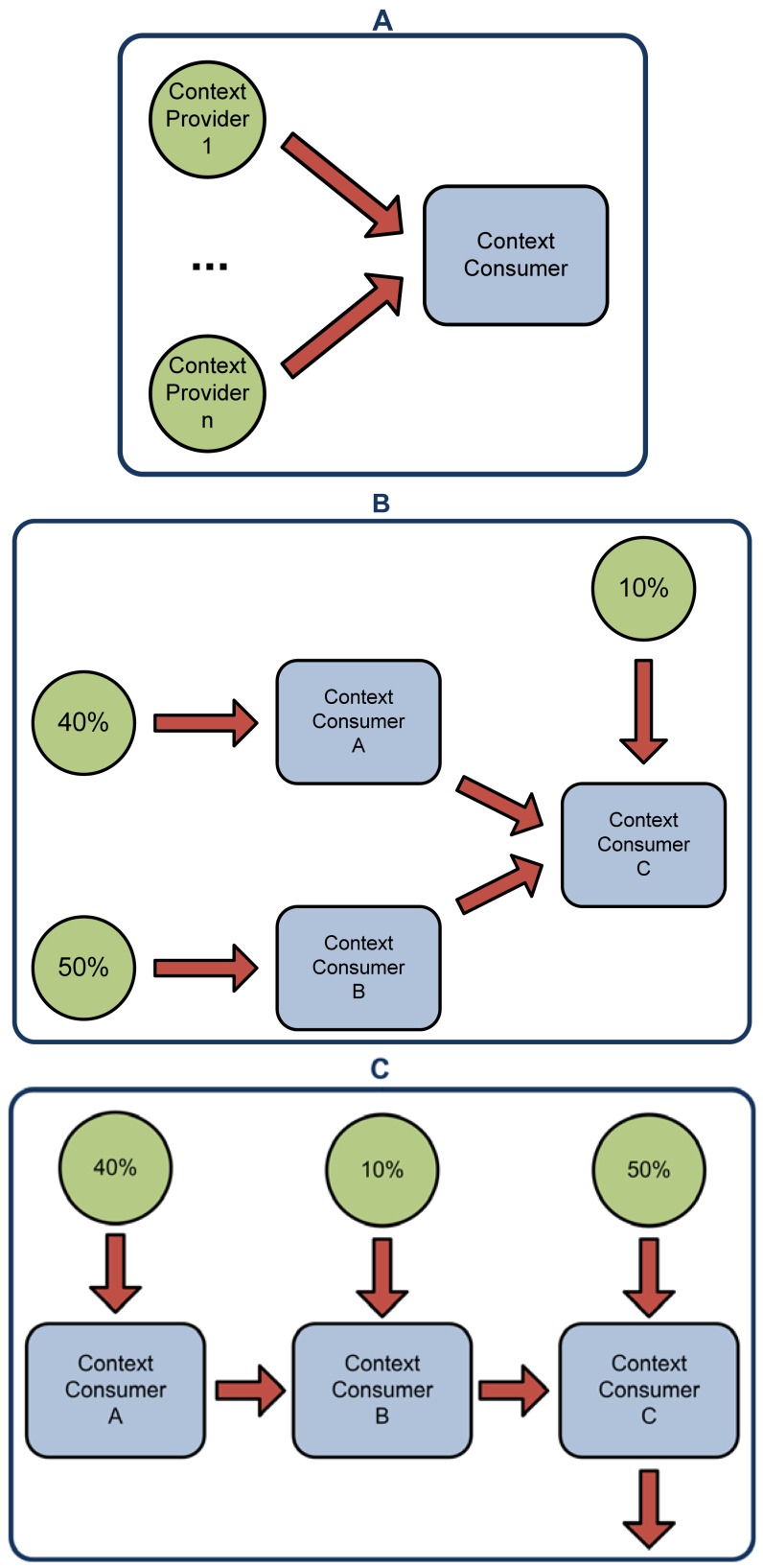

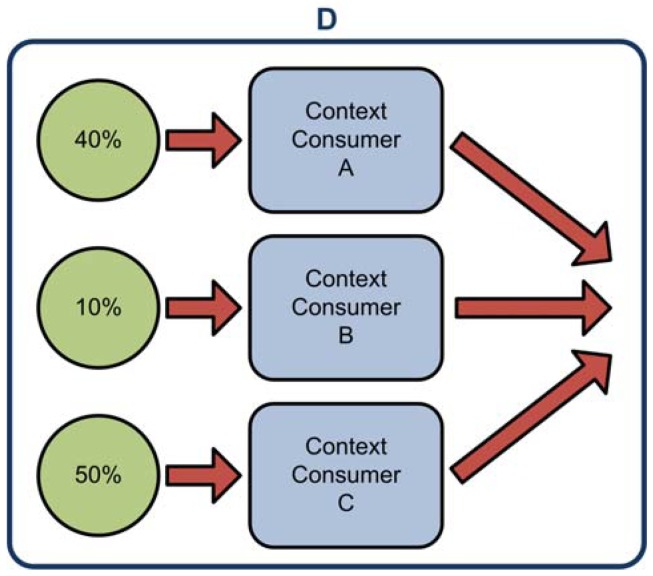
Scenarios for the evaluation of the system. First one (**A**) shows a centralized approach where a single inference engine processes all the data from the context providers; The second one (**B**) shows a distributed approach where three inference engines process the data from the context providers; The third one (**C**) is a completely serialized approach where the outcome of the previous reasoner is the input of the next one; Finally, the fourth scenario (**D**) shows a completely parallelized inference process.

To evaluate the developed system we have created four scenarios. In the first one a centralized inference engine processes all the data from the context providers (see Figure 8(A)). In the second one a distributed architecture of inference engines is used (see Figure 8(B)). This scenario has three inference engines (Context Consumer A, B and C). Some of the inference have been parallelized (Context Consumer A and B), while part of it can only be performed after some of the data has been processed (Context Consumer C). The context data has been distributed between the three Context Consumers. Context Consumer A will process the 40% of the Context Providers, Context Consumer B will process the 50% and Context Consumer C will take the final 10% of the Context Providers and the inferred facts from Context Consumer A and B. In the third scenario (see Figure 8(C)) the inference process is done in three serialized Context Consumers. The context data has been distributed between the three of them. Context Consumer A takes the 40% of the data, Context Consumer B takes the inferred facts from A and another 10% of the context data and finally Context Consumer C will process B's inferred facts and the other 50% of the context data. In the fourth and final scenario (see Figure 8(D)) Context Consumers process the context data at the same time in a completely parallel architecture. The data is divided in the same way it was divided in the third scenario.

A summary of the experiments can be found in Table 1:

- For each scenario we have performed a series of experiments using from 40 to 300 Context Providers.
- Each experiment has been repeated 100 times and the average time that took to resolve the complete inference problem was calculated.
- In all scenarios the inference engines were running in an Intel Core Duo P8700 with 4 GB of memory. As we explain in Section 6, currently is not possible to perform these tests in embedded devices.
- The used inference engine was the one provided with the Jena Framework [14].
- The average network latency during the experiments was of 50 ms. In the next subsection we analyze how the latency changes influence the results.
- The simulations used do not take into account node mobility or connectivity ranges. For the current state of the system we assume that nodes are static and that they do not disappear due to mobility or connectivity range problems.

**Figure 9.**
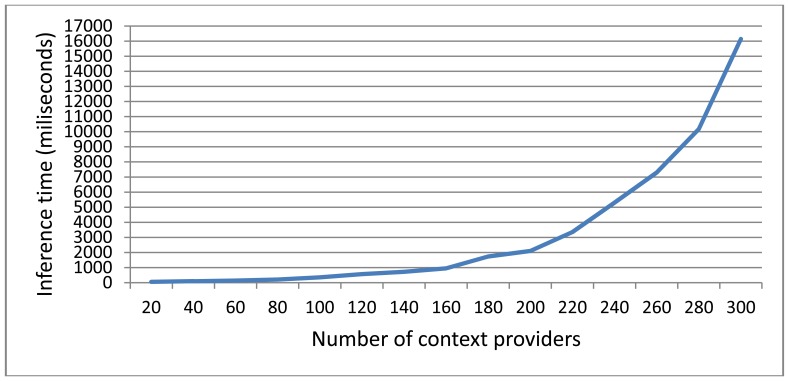
Inference times (in milliseconds) for a centralized approach where only one inference engine processes all the information from the Context Providers.

As can be seen in Figure 9 the centralized approach (Scenario A) inference time degrades drastically as the number of Context Providers (and hence the data to be processed) increases. As we discussed in the introduction the response time to context changed is an important factor in smart environments. Users need to see the reactions of to these changes to happen in a timely manner, otherwise the usability of the whole system will suffer.

**Figure 10.**
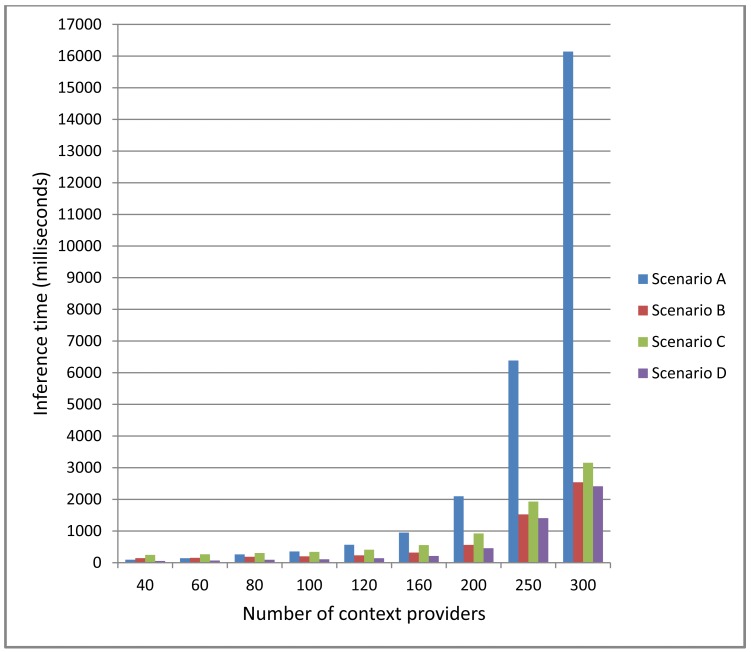
Comparison of inference times (in milliseconds) between the centralized and distributed approaches.

In Figure 10 it can be seen the differences between the centralized (Scenario A) and distributed (Scenario B, C and D) approaches. For a small number of context providers the centralized approach is much more efficient. The time gained parallelizing the inference process is minimal and much more time is lost due to the network latency. But as the number of Context Providers increases the distributed approach becomes more efficient. Even with 250 Context Providers the inference time for the distributed approach is under 2 s, while the inference time of the centralized approach is of 6,386 ms. This difference is even bigger with 300 Context Providers, where the centralized approach is five times slower.

The architecture of the distributed approaches will be dictated by the specific application domain. In order to evaluate the differences we have tested three different distributed architectures. A completely serialized architecture (Scenario C) where the output of each Context Consumer is the input of the next Context Consumer, an architecture where context data is processed in a completely parallel manner (Scenario D) and a scenario that mixes both approaches (Scenario B). As expected, among the distributed scenarios, D achieves the fastest inference times and C the slowest ones, being B's results between both of them. It has to been taken into account that Scenarios C and D are extremes of the possible distributed architectures and will not be usually encountered in real life problems. It is interesting to note that even the slowest distributed scenario performs better that the centralized one once the number of the Context Providers increases. As can be seen in Figure 9 the inference time increases exponentially with the number of Context Providers. As we discuss in the next sub-section, even if the process is completely serialized, splitting the inference problem helps reducing the global inference time.

### 5.1. Discussion

As result of the evaluation of the system we have identified four factors that influence the global performance of the system:

1. The hardware: Obviously the computational capabilities of the hardware where the inference is carried on will determine how fast that reasoning is performed.
2. The inference engine: The used semantic inference engine will also result in a faster or slower reasoning process. In [4] we analyzed the performance of different semantic reasoning engines.
3. The architecture of the reasoning engine ecosystem: The final architecture of the distributed reasoning ecosystem will depend on the specific domain of the problem. The architecture will dictate how much of the reasoning process can be parallelized.
4. The network latency: In the case of the distributed reasoning process the network latency is an important factor to take into account. The latency time must be added to the total inference time.

Points one and two are common for any reasoning architecture (centralized or distributed). The advantages or drawbacks that arise from the design choices in those features will be shared by both approaches. Points three and four are more interesting, because it is in those design choices where one approach will differ from the other. We will center our analysis in these last two factors. In the case of the architecture of the reasoning engine ecosystem it is important to design it to parallelize as much inference as possible. The distribution of the inference can be done according to three aspects as explained in Section 3: the location where the data is originated on, the context type expressed by the data and the certainty factor associated to the data. We acknowledge that there are scenarios where the restrictions imposed by the domain makes impossible to parallelize the inference process (e.g., if there is only one type of sensors in a single location), but usually smart environments are a host of a diverse ecosystem of sensors and devices and encompass a space divided in a series of rooms (a house, a building, an office, *etc.*). The next step is to analyze the “inference flow” and identify which sub-units of the inference problems can be parallelized and which ones depend on a previous inference, an example of this can be seen on Figure 1. Usually there are several inference sub-units in a problem than can be processed in parallel, reducing the total inference time for the whole system.

The second factor, network latency, penalizes the distribution of the inference problem. High network latency can make unfeasible to share the inference between multiple reasoning engines. It is easy to see how these two factors work against each another. On one hand we want to have as many inference sub-units as possible distributed among several reasoning engines. On the other one having multiple reasoning engines that have to communicate with each other can be counterproductive depending on the existing network latency. It is important to reach equilibrium between them to maximize the achieved improvement. This improvement can be expressed as:

(1) $$T_{\text{gain}}=T_{\text{par}}-T_{\text{lat}}$$

where:

- ***T_gain_*** is the total time gained distributing the inference.
- ***T_par_*** is the time gained with the parallelization of the different inference sub-units and the reduction of triples for each reasoning engine.
- ***T_lat_*** is the time lost in the communication between the reasoning engines due to the network latency.

It must be taken into account that other benefits arise also from the distribution of the reasoning process. The spatial decoupling assures that at least some of the inference will take place if some of the reasoning engine fails (due to connectivity problems, hardware failure). This increases the overall robustness of the system, making it more reliable. In the end, in order to decide if a distributed or centralized approach is more appropriate for a specific problem domain, users much ask themselves the following questions:

1. What is the average network latency?
2. Can the inference be parallelized or is a completely serial process in this domain?
3. How much semantic data must be processed? Only the measures from a few sensors or the data produced by all the users and sensors of a smart building?
4. Is important the robustness of the system? Partial solutions of the inference problem are useful?

### 5.2. Limitations of the System

The system has several limitations in its current state:

- There is no mechanism to automatically assess the certainty factor of the context providers. This process must be done manually by the user deploying the system, testing each sensor type to evaluate the uncertainty in their measures.
- The computational capabilities of each device are not taken into account in the negotiation process. This can result in computationally constrained devices been burdened with a large fraction of the inference problem.
- Related with the previous point, the system does not allow to dynamically reassign the rules from one reasoning engine to another according to their capabilities.

We are working on addressing these limitations in future versions of the systems. Implementing these characteristics will lead to a more robust system that can adapt itself to the devices present in the environment for a more efficient reasoning process.

## 6. Conclusions and Future Work

In this paper we have discussed the problems of semantic inference in smart environments. On one hand ontologies are one of the best approaches to context-modeling. On the other hand the semantic reasoning can be slow and cumbersome. In order to tackle this problem in smart environments with a rich ecosystem of computationally enabled objects and devices we propose to divide the inference process into smaller units and distribute it among several semantic inference engines. Distributing the inference process we attain several advantages. First, we obtain temporal and spatial decoupling of the inference sub-units. This is done splitting the reasoning problem into different agents of the network. Thanks to the spatial decoupling, if one node loses network connectivity or suffers a hardware problem the others can try and partially solve the reasoning problem without the output of that inference sub-unit. Depending on the problem domain the reasoning engine network will be able to attain a partial solution instead of no solution at all. The temporal decoupling allows us to parallelize the inference, reducing the total time needed to achieve a solution (see Section 5 and Figure 8). Second, we reduce the number of triples that each reasoning engine has to process by sharing then among the different agents in the network. As seen in Figure 9 and explained in [4], the inference time increases exponentially as the data to be processed does. Splitting the data between different agents helps us, along with the temporal decoupling, to reduce the total inference time. Third we compartmentalize the information in different reasoning engines depending on the interests of each one. Currently our system does not have any security mechanism that takes advantage of this characteristic to enforce privacy settings on context data, but is an interesting feature that we plan to implement in the future. Finally due to the negotiation mechanism the reasoning engine architecture can reorganize itself if there is any change in the reasoning engines interests or the nature of the context providers. This allows our system to adapt to the changes in the nature of the environment.

The main problem to deploy the system in real environments is running the existing semantic reasoning engines into embedded devices. As already stated by Seitz and Schönfelder [32] none of the existing semantic reasoning engines can be used in embedded platforms. In order to solve this problem we are currently developing a similar semantic reasoning engine, also based in CLIPS [33], which implements the OWL 2 RL [34] profile. This reasoning engine will be able to run not only in embedded Linux platforms but also on Android based systems. On the other hand there are several examples of the Jade framework running in portable and mobile platforms like J2ME and Android, so once the semantic reasoning engine is finished we plan to perform a more in-depth test using more computationally constrained devices. The results in more computationally limited devices will surely produce higher inference times, but it will have the same effect in the centralized (only one reasoning engine doing all the inference) and distributed (using our system to split the reasoning problem between different reasoning engines) approaches. Thus, the results obtained in the validation section can be used as an approximation to the expected results in other platforms.

As future work we intend to create a more complex negotiation process where the computational capabilities of each device are taken into account to better divide the inference according to them. This will allow us to create a more efficient reasoning architecture that will improve the global inference time. We would also like to dynamically reassign the rules from one reasoning engine to another according to their capabilities. To do this we intend to automatically map the “inference flow” of the domain, identifying those inference sub-units that can be parallelized and organizing the rules so that we can achieve the maximum efficiency.

## Figures and Tables

**Table 1.** Configuration of the experiments.

**Scenario**	**Approach**	**# Of Context Providers**	**Network Latency (ms)**	**Context Consumer's Hardware**	**# Of Experiments Performed**
**A**	Centralized	40, 60, 80, 100, 120, 160, 200, 250, 300	50	Intel Core Duo P8700 with 4 GB of memory	100
**B**	Distributed (mixed)				
**C**	Distributed (serialized)				
**D**	Distributed (parallel)				
